# Early Access for Medicines in ITALY: The Case of Ruxolitinib for Patients with Graft-Versus-Host Disease

**DOI:** 10.3390/jcm13144273

**Published:** 2024-07-22

**Authors:** Lucia Gozzo, Salvatore Leotta, Giovanni Luca Romano, Calogero Vetro, Andrea Duminuco, Giuseppe Milone, Alessandra Cupri, Fanny Erika Palumbo, Serena Brancati, Rosy Ruscica, Laura Longo, Daniela Cristina Vitale, Giorgia Fiorenza, Giovanni Enrico Lombardo, Antonio Lazzara, Francesco Di Raimondo, Giuseppe Alberto Palumbo, Filippo Drago

**Affiliations:** 1 Clinical Pharmacology Unit/Regional Pharmacovigilance Centre, University Hospital of Catania, 95125 Catania, Italy; serena.brancati@gmail.com (S.B.); rosyruscica@gmail.com (R.R.); longolaura@hotmail.com (L.L.); danielac.vitale@gmail.com (D.C.V.); f.drago@unict.it (F.D.); 2 Haematology and BMT Unit, University Hospital of Catania, 95125 Catania, Italy; leotta3@yahoo.it (S.L.); gerovetro@gmail.com (C.V.); andrea.duminuco@gmail.com (A.D.); giuseppe.milone@gmail.com (G.M.); alessandracupri@hotmail.it (A.C.); fannypalumbo@gmail.com (F.E.P.); diraimon@unict.it (F.D.R.); palumbo.giuseppealberto@gmail.com (G.A.P.); 3 Faculty of Medicine and Surgery, University of Enna “Kore”, Piazza dell’Università, 94100 Enna, Italy; giovannienrico.lombardo@unikore.it; 4 Department of Biomedical and Biotechnological Sciences, University of Catania, 95125 Catania, Italy; giorgia.f1d@gmail.com; 5 Health Department, University Hospital of Catania, 95125 Catania, Italy; antonio.lazzara@gmail.com; 6 Centre for Research and Consultancy in HTA and Drug Regulatory Affairs (CERD), University of Catania, 95125 Catania, Italy

**Keywords:** ruxolitinib, graft-versus-host disease, access, health technology assessment

## Abstract

After European Medicines Agency (EMA) approval, national pricing and reimbursement procedures are necessary to guarantee access to drugs, based on the willingness to pay and the recognition of therapeutic value. These can result in delays in drug availability for patients, even for those with important unfmet needs for whom it may be necessary and ethical to ensure access. The objective of this study was to evaluate the use of ruxolitinib for patients with graft-versus-host disease (GvHD) after EMA approval at the University Hospital of Catania. We analysed data about the use of ruxolitinib in patients with GvHD, describing their basic characteristics, their outcomes and the cost of the treatment. In the reference period, 24 ruxolitinib treatments were started according to the Summary of Product Characteristic. The average treatment duration was 10 months. Twenty patients showed a response, maintained over time, with no adverse reactions. The total expenditure amounts to EUR 963,424. The use of ruxolitinib in a real population confirms its role in an important therapeutic need. The quantification of costs requires a reflection on the sustainability of early access to medicines authorised by the EMA for serious diseases and in the absence of therapeutic alternatives.

## 1. Introduction

Allogeneic haematopoietic stem cell transplantation (alloSCT) is a potentially curative treatment option for malignant and non-malignant haematological diseases [1]. However, treatment-associated graft-versus-host disease (GvHD) remains a challenge of alloSCT, limiting the possibility of obtaining successful transplant outcomes. Indeed, it is the most frequent cause of non-relapse morbidity and mortality, developing in up to 70% of transplant recipients [2,3,4,5,6].

It may occur in an acute form (aGvHD) and a chronic form (cGvHD) [7], with multiorgan involvement (e.g., skin, liver, lower and upper gastrointestinal tract, eye, and lung).

Immunosuppressive regimens, including calcineurin inhibitors (CNI), methotrexate (MTX), mycophenolate mofetil (MMF), anti-thymocyte globulin (ATG), rituximab or T-cell depletion [8,9], represent the most common preventive methods, although treatments can vary across institutions. With regard to treatment, it is based on the severity of symptoms and the organs affected. Topical corticosteroids or CNI are recommended for patients with grade I aGvHD and mild cGvHD localised to the skin [10,11], whereas systemic corticosteroids are the standard first-line treatment for grade II to IV aGvHD and moderate-severe cGvHD, but less than 50% of patients obtain a durable response and become steroid resistant/refractory [12,13,14,15,16].

Patients with steroid-refractory disease (SR-GvHD) have a poor prognosis and a high risk of mortality with a 6-month survival for SR-aGVHD of approximately 50%, and a 2-year survival of 30% or less [17].

These patients require the administration of second-line treatments, still not uniformly recognised [18,19].

The most common agents recommended in SR-GvHD include ruxolitinib, extracorporeal photopheresis (ECP), MTX, MMF, mTOR inhibitors (everolimus or sirolimus), or infliximab, used alone or in combinations with steroids [9,12,16].

The European Society for Blood and Marrow Transplantation (EBMT) recommends ruxolitinib in adults with SR-aGVHD and SR-cGVHD, due to its large beneficial effect demonstrated in randomised trials and meta-analyses without relevant adverse events (AEs) [20].

Ruxolitinib is a potent and selective inhibitor of Janus Kinases (JAKs) developed for symptomatic myelofibrosis and polycythemia vera [21,22,23], which has been recently approved for acute and chronic SR-GvHD in patients aged 12 years and older who have inadequate responses to corticosteroids or other systemic therapies [24,25].

An open-label, randomised trial showed an overall response advantage and a superior failure-free survival in favour of ruxolitinib with respect to the best available therapy in patients with glucocorticoid-refractory or dependent systemic chronic GVHD [26], and in 2021 the FDA approved ruxolitinib for the treatment of cGVHD after the failure of one or more lines of systemic therapy [27].

The drug demonstrated a statistically significant higher overall response rate [ORR = complete response (CR) + partial response (PR)] compared to the control for SR-aGvHD (62% versus 39% at day 28; odds ratio [HR] 2.64; *p* < 0.0001) and SR-cGvHD (49% versus 25% at cycle 7; HR 2.99; *p* < 0.0001) [25]. In general, CR rates were low. The most frequent AEs were infections and infestations, and blood and lymphatic system disorders.

In April 2022, the European Medicines Agency (EMA) issued a positive opinion on the extension of indication of ruxolitinib in this population. However, after EMA approval, national pricing and reimbursement procedures are necessary to guarantee access to drugs, based on the willingness to pay and the recognition of therapeutic value [28,29,30].

In Italy this procedure is still ongoing (updated June 2024), resulting in a delay in ruxolitinib reimbursement and availability for patients. Nevertheless, under certain circumstances, it is possible to obtain a drug before the conclusion of price and reimbursement agreements for those with an important unmet need for whom it may be necessary and ethical to ensure access as soon as possible. For example, this can be allowed through compassionate use in the event that the company agrees to freely supply the drug. In the case of ruxolitinib, a compassionate use programme is not available, and the drug can only be funded by health facilities, without national health system (NHS) reimbursement.

This study aimed to evaluate the use of ruxolitinib for patients with GvHD at the University Hospital of Catania after EMA approval and before the conclusion of the negotiation process.

## 2. Materials and Methods

We analysed data collected by the Regional Pharmacovigilance Centre as part of the evaluation of prescriptions made by clinicians to obtain the authorisation of the Hospital Health Director to purchase medicines that were not reimbursed, based on the therapeutic needs and the availability of therapeutic alternatives for each patient. In the event of a positive assessment, a specific authorisation is issued per patient, to be renewed every 3 months, according to the treatment response at follow-up.

Specifically, the use of ruxolitinib was evaluated in patients aged 12 and older with aGvHD or cGvHD and inadequate response to corticosteroids or other therapies, according to EMA authorisation.

Patients treated from April 2022 (the date of EMA approval) to April 2024 for at least 3 months were included.

The severity of acute and chronic GVHD was graded according to the Mount Sinai International Consortium on aGVHD (MAGIC) criteria [31]. Chronic GVHD was diagnosed and scored according to the National Institute of Health (NIH)-defined criteria [7] while the response to the treatment was assessed according to the NIH-response criteria [32]. A descriptive analysis was performed, describing the baseline characteristics of patients, the treatments used for GvHD, the outcome and the costs of ruxolitinib. The ‘time to response’ has been defined according to the first available follow-up report, required for the renewal of the authorisation issued by the Hospital Health Director.

## 3. Results

In the reference period, a treatment with ruxolitinib was started in 24 patients with a mean age of 53 (range 35–71), comprising 15 males and 9 females, 16 with cGvHD and 8 with aGvHD, after transplantation from a matched related (*n* = 16) or unrelated donor (*n* = 8), as shown in Table 1. Patients had on average three organs involved (range 2–5), namely skin (*n* = 23), mucosae (*n* = 14), eye (*n* = 10), gastrointestinal tract (*n* = 12), liver (*n* = 8), lung (*n* = 3), muscle (*n* = 2) and reproductive organs (*n* = 1), and had received at least three lines of therapy for GvHD (mean 4, range 3–6), including corticosteroids, ECP, anti-lymphocytic serum and CNI.

The drug was used according to the recommendations of the Summary of Product Characteristic (SmPC) at a dosage of 20 mg/die for 10 months (range 3–21; Table 2). Twenty patients (83.3%) showed a response to the treatment, of which 12 were complete (50%) and 8 were partial responses (33%), maintained over time (median duration of response = 11 months, range 1–18). For three patients in CR the discontinuation of the drug led to the reactivation of the disease.

Data were not available for two patients (8.2%) lost to follow-up. Two patients showed progressive disease (PD) and one of them died within 100 days of transplantation because of sepsis due to carbapenemase-producing Klebsiella pneumoniae. No adverse drug reactions were reported.

The cost of 10 months of therapy was EUR 41,188/patient (ex-factory price of 10 mg tablets, 56 tablets = EUR 4188.8). The total expenditure to date amounts to EUR 963,424.

## 4. Discussion

In accordance with European regulations, medicines containing a new active substance (and new therapeutic indications) need to be approved by the EMA through the centralised procedure in order to be marketed [33]. However, after EMA approval, each country is responsible for national market access, following the assessment of the therapeutic value and the price and reimbursement agreement definition. [28,29,30]. This process may take a long time to be completed, resulting in issues and delays in terms of access [34].

Moreover, it has been demonstrated that the time to reimbursement (TTR) of new medicines (or new indications) differs among countries based on several factors, including among others the supporting evidence, the level of innovation, the product price and the gross domestic product (GDP) of the country [35,36,37,38,39,40,41,42].

A previous study on data from some European national authorities found a TTR of 407 days (median; range -81-2320 days) for oncologic drugs approved in Europe from 2016 until 2021. The TTR was significantly different among countries, although the Council of European Communities has set 180 days as the deadline for member states to conclude pricing and reimbursement procedures, particularly for innovative medicines [43]. The shortest TTR has been identified for Germany (median 3 days), in line with the practice of setting a free price at market launch followed by a negotiation using various parameters, including the added therapeutic value [44,45].

According to the latest report released by the Italian Medicines Agency (AIFA), in Italy the time between EMA approval and the conclusion of the national procedures is equal to on average 273.1 days, without considering the time for the submission of the dossier and the time until its publication in the AIFA Official Journal [46]. This can be related to uncertainties in terms of efficacy and safety making it difficult to define the therapeutic value of the drug based on the available data. Furthermore, a lack of agreement on the price and reimbursement conditions between companies and payers may lead to a long negotiation time.

The use of ruxolitinib in patients with GvHD was approved by the EMA in April 2022, although it is not reimbursed yet in Italy after more than 2 years (updated June 2024), despite the high unmet need of this difficult-to-treat population. The reasons for this delay are not known.

It is noteworthy that, in December 2022, the AIFA issued a positive opinion on the reimbursement of the drug according to Law 648/96, perhaps to anticipate access to the drug pending the conclusion of the price and reimbursement procedures [47]. Indeed, this Italian law allows the use of innovative medicines authorised in other countries but not in Italy, medicines under clinical trial and off-label drugs [48,49,50]. However, this opinion has never entered into force.

On the contrary, in March 2022 the French National Authority (Haute Autorité de santé, HAS) granted an early access authorisation for the use of ruxolitinib in patients with GvHD as the disease is serious, rare and disabling, and there is no appropriate treatment [51]. The authorisation was valid until the decision to reimburse the drug [52].

The drug demonstrated a high response rate in clinical trials, confirmed by meta-analyses of the available evidence, without significant risks [26,53,54,55,56,57,58,59]. Favourable results in terms of efficacy and safety have been shown in both acute and chronic GvHD, including high-risk diseases such as gut III-IV aGvHD and lung GvHD.

Since a compassionate use programme is not available, to ensure access for patients eligible for treatment in the absence of therapeutic alternatives the drug can be funded in Italy only by the health facilities directly. Thus, the costs of these treatments must be covered by hospitals’ own funds.

At the University Hospital of Catania, the Hospital Health Director is responsible for the authorisation of the supply of not reimbursed medicines, based on the case-by-case assessment of therapeutic needs and the availability of therapeutic alternatives carried out with the support of clinical pharmacologists. The treatment costs are also included in the assessment, taking into account the need to control pharmaceutical expenditure to avoid an excessive burden on the hospital budget.

In this regard, in the two years after EMA approval 24 treatments have been started following prescription by clinicians and assessment by pharmacologists. More than 80% of patients showed a response to treatment, with a high percentage of CR (50%), maintained over time, in line with the available evidence. The management of responders includes the gradual reduction of dosage and the drug discontinuation if the response is maintained. We observed a GvHD reoccurrence in 3/5 patients who discontinued ruxolitinib after a long-lasting CR, and the treatment was restarted.

These positive results confirm the role of ruxolitinib in this clinical setting, and the importance of accelerating the reimbursement decision to make the drug available for all eligible patients.

However, the choice of guaranteeing this and other treatments not reimbursed poses issues related to the sustainability of their costs, if we consider the significant increase in the hospital pharmaceutical expenditure.

This study underlines the importance of facing the challenge of providing quick and sustainable access to and reimbursement of new medicines (or new indications of old medicines).

A novel and specific regulatory model should be introduced to guarantee immediate access after centralised approval has been granted for drugs which could represent a real innovation for the management of patients with relevant unmet needs, as well as to streamline the national decision-making process, without compromising the sustainability for the NHS.

In this way, patients who are considered eligible for treatment would benefit from the therapy immediately and without delay.

## 5. Conclusions

Ruxolitinib has changed the management of SR-GvHD, becoming the drug of choice after the failure of corticosteroids.

The analysis of data in a real population confirms the role of the drug in patients with an important therapeutic need.

The quantification of costs, however, requires a reflection on the sustainability of early access to medicines authorised by the EMA for serious diseases and in the absence of effective therapeutic alternatives.

Current early access tools proved to be insufficient in most cases to meet these needs. Therefore, it is necessary to find alternative methods to cover important therapeutic needs with rapid and sustainable access as well as to accelerate the negotiation process in general.

## Figures and Tables

**Table 1:** Baseline characteristics of patients. ALL = acute lymphoblastic leukemia; AML = acute myeloid leukemia; CML = chronic myeloid leukemia; CMML = chronic myelomonocytic leukemia; MDS = myelodysplastic syndrome; MM = multiple myeloma; MRD = matched related donor; MUD = matched unrelated donor; PI = primary immunodeficiency; PRCA = pure red cell aplasia.

Patient	Age	Gender	Disease	Underlying Disease	Transplant	Donor	Organs Involved	Previous Treatments
1	40	M	cGVHD	PI	September 2019	MRD	4	4
2	63	M	cGVHD	MDS	November 2020	MRD	4	4
3	67	M	cGVHD	CMML	July 2020	MRD	4	4
4	49	M	cGVHD	MDS	June 2020	MRD	4	4
5	36	M	cGVHD	AML	September 2018	MRD	4	4
6	59	M	cGVHD	AML	September 2018	MRD	2	4
7	53	F	cGVHD	ALL	July 2007	MRD	3	6
8	62	M	cGVHD	AML	2016	MUD	4	4
9	41	F	cGVHD	AML	June 2019	MUD	4	5
10	65	M	aGVHD	PRCA	October 2022	MRD	2	4
11	50	M	aGVHD	AML	March 2022	MRD	3	4
12	35	M	cGVHD	AML	December 2020	MUD	5	4
13	48	M	aGVHD	MDS	January 2023	MRD	3	4
14	49	F	aGVHD	MDS	January 2023	MRD	2	4
15	60	F	cGVHD	MM	January 2016	MUD	4	5
16	55	F	cGVHD	AML	February 2022	MRD	2	4
17	44	F	cGVHD	AML	September 2022	MUD	2	4
18	57	M	cGVHD	Lymphoma	October 2020	MRD	3	4
19	71	F	aGVHD	MDS	May 2023	MRD	2	4
20	60	M	cGVHD	MDS	February 2022	MUD	3	4
21	61	M	aGVHD	AML	July 2023	MUD	2	3
22	38	F	cGVHD	CML	July 2023	MUD	3	5
23	54	F	aGVHD	Lymphoma	October 2023	MRD	2	3
24	50	M	aGVHD	Lymphoma	October 2023	MRD	2	3

**Table 2:** Treatment characteristics and outcome.

Patient	First Prescription	Treatment Duration (months)	Response	Time to Response (months) ^a^	Loss of Response	Duration of Response (months)	Discontinuation
1	July 2022	21	CR	3	/	18	No
2	July 2022	21	CR	3	/	18	No
3	July 2022	15	CR	3	/	18	Yes
4	August 2022	21	CR	3	/	18	No
5	August 2022	15	PR	3	/	18	Yes
6	October 2022	16	CR	3	Reactivation *	13	No **
7	November 2022	3	PR	3	/	/	Yes
8	November 2022	17	PR	3	/	14	No
9	November 2022	17	CR	3	/	14	No
10	December 2022	9	PR	3	/	6 ^b^	Yes
11	December 2022	6	CR	3	/	13	Yes
12	February 2023	14	PR	3	/	11	No
13	March 2023	3	PD-Death	/	/	/	No
14	March 2023	12	CR	3	Reactivation *	9	No ***
15	May 2023	11	PR	3	/	8	No
16	June 2023	7	CR	3	Reactivation *	4	No ***
17	July 2023	3	NA ^b^	/	/	/	/
18	July 2023	9	CR	3	/	6	No
19	July 2023	9	CR	4	/	5	No
20	September 2023	8	PR	3	/	5	No
21	December 2023	3	NA ^a^	/	/	/	/
22	December 2023	5	CR	3	/	1	No
23	January 2024	4	PR	3	/	1	No
24	January 2024	3	PD	3	/	/	Yes

^a^ defined according to the first available follow-up report; * reactivation after drug discontinuation; ** re-treatment due to reactivation after drug discontinuation on February 2024; *** re-treatment due to reactivation after drug discontinuation on March 2024; ^b^ lost to follow-up; CR = complete response; NA = not available; PR = partial response; PD = progressive disease.

## Data Availability

Dataset available on request from the authors.
